# Genotype analysis of polymorphisms in autoimmune susceptibility genes, *CTLA-4* and *PTPN22*, in an acute anterior uveitis cohort

**Published:** 2009-01-26

**Authors:** Tammy M. Martin, Louise Bye, Neil Modi, Miles R. Stanford, Robert Vaughan, Justine R. Smith, N. Kevin Wade, Friederike Mackensen, Eric B. Suhler, James T. Rosenbaum, Graham R. Wallace

**Affiliations:** 1 Oregon Health & Science University, Portland, OR; 2 King's College, London, UK; 3 Kerrisdale Professional Centre, Vancouver, British Columbia, Canada; 4 University of Heidelberg, Heidelberg, Germany; 5 Portland Veterans Affairs Medical Center, Portland, OR; 6 University of Birmingham, Birmingham, UK

## Abstract

**Purpose:**

Acute anterior uveitis (AAU) is the most common form of uveitis and is thought to be autoimmune in nature. Recent studies have described genes that act as master controllers of autoimmunity. Protein tyrosine phosphatase type 22 (*PTPN22*) and Cytotoxic T lymphocyte antigen-4 (*CTLA-4*) are two of these genes, and single nucleotide polymorphisms (SNPs) in the genes encoding these molecules have been associated with several autoimmune diseases. In this study we have analyzed SNPs in *PTPN22* and *CTLA-4* in patients with AAU.

**Methods:**

The functional protein tyrosine phosphatase type 22 (*PTPN22*) SNP (R620W rs2476601, 1858C/T), and two *CTLA-4* SNPs (rs5742909, −318C/T and rs231775, 49A/G) were analyzed in 140 patients with AAU and 92 healthy controls by sequence-specific primer -polymerase chain reaction (SSP-PCR). Data was analyzed by χ^2^ analysis and Fisher’s exact test.

**Results:**

There was no significant association between *PTPN22* 620W, *CTLA-4* −318C/T, or *CTLA-4* 49A/G and AAU. Similarly, there was no association with the three SNPs when patients were classified by race or gender. Finally, there was no association with the presence of ankylosing spondylitis in the patient cohort.

**Conclusions:**

The data do not support an association between SNPs in *PTPN22* and *CTLA-4*, genes regarded as genetic master switches of autoimmunity. This raises the issue of the etiology of AAU and the possibility that it should be regarded as an autoinflammatory rather than an autoimmune condition.

## Introduction

Uveitis or inflammation of the uvea is a significant cause of visual loss. Clinically, uveitis can be categorized into distinct phenotypes. Acute anterior uveitis (AAU), which presents unilaterally with sudden onset, is self-limiting and recurrent and represents the specific uveitis phenotype associated with ankylosing spondylitis (AS) and other seronegative spondyloarthropathies (SpA) [1]. A link between AAU, AS, and SpA is the major histocompatability complex (MHC) class I molecule, human leucocyte antigen B-27 (HLA-B*27). Although the frequency of patients with AAU who are positive for HLA-B*27 varies among different published surveys, it is generally estimated that among Caucasian patients with AAU, 50% are positive for HLA-B*27 [2].

It is clear that even in HLA-B27-associated AAU, other genetic factors are involved. Several functional polymorphisms in genes that produce proteins relevant to the immune response have been described to either confer an altered susceptibility to autoimmune diseases or appear to influence their severity and outcome. Identification of such polymorphisms is important as it may help to advise patients on prognosis and influence the choice and dose of immunosuppressive drugs, particularly biological agents [3,4].

A functional single nucleotide polymorphism (SNP) of the gene encoding protein tyrosine phosphatase type 22 (*PTPN22*; R620W rs2476601, 1858C/T) has recently been described as a strong common genetic risk factor for human autoimmune disease [5]. *PTPN22* is located on chromosome 1p13.3 and encodes a lymphoid-specific phosphatase (Lyp) that binds to c-src tyrosine kinase (Csk), an intracellular tyrosine kinase. Csk phosphorylates leucocyte-specific tyrosine kinase (Lck), leading to the inhibition of Lck kinase activity. The PTPN22 R620W mutation leads to reduced binding of Lyp to Csk, resulting in reduced Lck inactivation, with the overall effect of a gain of function mutation. This is associated with downregulation of early T cell receptor signaling that results in an increase in autoreactive T cells and autoantibody production, a process thought to be due to defective deletion of autoreactive T cells during thymic maturation or dysfunction of regulatory T cells [6]. PTPN22 R620W has been associated with rheumatoid arthritis, type 1 diabetes mellitus, systemic lupus erythematosus, and autoimmune thyroiditis. In contrast, PTPN22 R620W has not been associated with two other presumed autoimmune disorders, multiple sclerosis and Crohn’s disease [7,8].

Human Cytotoxic T lymphocyte antigen-4 (*CTLA-4*) is located on chromosome 2p33, and two well studied polymorphisms in this gene are −318 C to T, (rs5742909) and −49 A to G (rs231775). In particular, the 49G polymorphism has been linked to reduced expression of CTLA-4 on the T cell surface, reduced soluble CTLA-4 production, and subsequently impaired inhibitory function [9]. The biological importance of such a change is supported by the findings that the 49 A/G SNP has been associated with Graves’ disease, type 1 diabetes mellitus, primary biliary cirrhosis, and, in combination with a second *CTLA-4* SNP 6230 G/A, an increased rate of acute rejection in patients undergoing liver transplantation [10-12]. In this study, PTPN22 R620W, *CTLA-4* −318C/T, and *CTLA-4* 49A/G were tested for association with disease in a cohort of patients with AAU. Associations with race, gender, and presence of AS were also tested.

## Methods

### Subjects

One hundred and forty subjects with AAU were recruited through Oregon Health and Science University, Portland, OR. The diagnostic validation of AAU was based on an ophthalmology chart review by J.R.S. The clinical criteria for the diagnosis of AAU include documented evidence through an assessment by slit-lamp biomicroscopic examination of anterior chamber inflammation that is sudden in onset, unilateral, and resolves within 12 weeks. This form of uveitis is typically episodic, and although it presents unilaterally, it may flip-flop, involving the companion eye in recurrence. Individuals were considered to be affected with uveitis if (1) they met the AAU criteria after careful review of ophthalmology chart notes or (2) they were referred to the study by a uveitis specialist with first-hand knowledge of the subjects’ uveitis presentation. Ninety-two race and gender matched healthy individuals were used as controls. This research was conducted under human subject protocols approved by the OHSU, the St. Thomas' Hospital, London and the Sandwell and West Birmingham National health Service Trust, Birmingham, UK local ethics committees and adhered to the Declaration of Helsinki agreement.

### Single nucleotide polymorphism analysis

After informed consent, blood samples were collected by venepuncture. Genomic DNA was prepared using standard salt extraction techniques and stored at –70 °C until use. The *PTPN22* R620W, *CTLA-4* −318C/T, and *CTLA-4* +49A/G SNPs were detected by sequence-specific primer-polymerase chain reaction (SSP-PCR) using primer mixes (Table 1).

**Table 1:** PCR primer sequences and length of amplification product.

**Gene**	**Allele**	**Sequence 5′-3′**	**Control primer**
*CTLA4*	−318C	CACTTAGTTATCCAGATCCTC	TGCCAAGTGGAGCACCCA A
	−318T	CACTTAGTTATCCAGATCCTT	GCATCTTGCTCTGTGCAGAT
	49A	CAGGGCCAGGTCCTGGT	
	49G	CAGGGCCAGGTCCTGGC	Product 745 basepairs
		Product 402 basepairs	

### Statistics

Associations with disease were sought between allele frequencies. χ^2^ analysis was performed using EpiStat (EpiStat Group Inc, Atlanta GA). A case-control analysis was employed using the Fisher’s exact test.

## Results

The AAU cohort consisted of 140 subjects (78 female, 62 male) with 133 identified Caucasian by self-report. Of the 140 with AAU, 76 were without history of AS while 58 had a diagnosis and the remaining six subjects were considered to have probable or early signs of AS. The healthy control cohort represented 92 subjects (54 female, 38 male), 81 of whom were Caucasian.

The minor allele frequencies of *CTLA-4* −318 and +49 were 8.9% and 42.1%, respectively, in the AAU group, and 4.9% and 36.4%, respectively, in the healthy controls. Although we noted slightly higher minor allele frequencies of both SNPs in the AAU group, the differences were not statistically significant (Table 2). The SNP allele frequencies in the control group were in agreement with other published Caucasian cohorts [9]. Furthermore, sub-analyses with only the Caucasian samples or only the subjects without AS or a haplotype analysis of the two SNPs (Table 2) did not reveal any significant differences between the cases and controls.

**Table 2:** Allele frequency and genotype analysis of *CTLA-4* −318 and +49 polymorphisms in patients with AAU and healthy controls.

	**AAU patients (n=140)**	**Controls (n=92)**	**p value**
*CTLA-4 −318*			
C	0.91	0.95	0.14
T	0.09	0.05	
Genotype			
CC	123	83	ns
CT	15	9	
TT	2	0	
*CTLA-4 +49*			
A	0.58	0.64	0.24
G	0.42	0.36	
Genotype			
AA	49	38	ns
AG	64	41	
GG	27	13	
*CTLA-4* Haplotype			
CA	138	108	0.06
CG	117	67	0.29
TA	25	9	0.15
TT	0	0	-

There was no significant differences in haplotype or genotype frequency between patients with AAU and healthy controls. ns=not significant.

The *PTPN22* R620W variant allele frequency was 9.8% in the healthy control cohort, which was similar to a UK control cohort and other published reports [7,13]. This was higher than the variant allele frequency found in the AAU group (6.5%), but this difference was not statistically significant (p=0.217; Table 3). By restricting the analysis to Caucasian samples, the variant allele frequency was 11.1% and 6.8% in controls and AAU patients, respectively (p=0.151). Furthermore, sub-analysis of only those AAU subjects without AS revealed no difference between controls (9.8%) and cases (9.21%, p=1.000).

**Table 3:** Allele frequency and genotype analysis of *PTPN22* 620W polymorphism in patients with AAU and healthy controls.

***PTPN22***	**AAU patients (n=140)**	**Controls (n=92)**	**p value**
Allele Frequency			
C	0.93	0.90	0.2
T	0.07	0.10	
Genotypes			
CC	123	75	ns
CT	14	16	
TT	2	1	

There was no significant differences in haplotype or genotype frequency between patients with AAU and healthy controls. ns=not significant.

## Discussion

Acute anterior uveitis (AAU) is the most common form of immune-mediated uveitis and is strongly associated with HLA-B27 and spondyloarthritis. In this study, we sought to determine if polymorphisms in genes encoding genetic master switches, *PTPN22* and *CTLA-4*, contribute to AAU. The results show that neither *PTPN22* R620W nor *CTLA-4* SNPs (−318, +49) are associated with AAU in this cohort. As the *PTPN22* 620W frequency varies in different geographical areas, we analyzed only the Caucasians in our cohort and again found no significant association. Finally, there was no association between any of the polymorphisms tested and AS in the patient group. These data show that *PTPN22* and *CTLA-4* polymorphisms do not influence onset or severity of AAU.

As stated, AAU has been strongly linked to HLA-B*27 and SpA. In recent studies, uveitis has been described as a first symptom in nearly half of SpA patients [14]. AAU has also been associated with early onset of SpA in HLA-B*27-positive patients [15]. Conversely, patients with an extraocular disease have a greater number of uveitis relapses compared to ocular disease alone. These studies involved patients at tertiary referral centers. By comparison, in a primary care setting, HLA-B*27, AS, or family history of AS did not influence relapses in these patients whereas patients with one relapse were more likely to have a second event [16]. Therefore, HLA-B*27 appears to be involved with more severe AAU.

AAU has been classified as an autoimmune condition. However, the evidence to support this is lacking. Numerous studies have shown serum antibodies to Gram negative bacteria, including *Helicobacter pylori*, *Chlamydia trachomatis*, *Salmonella*, and *Proteus* sp, in HLA-B*27–positive and SpA patients. In particular, the response to *H. pylori* was greater in patients compared to healthy controls, although this may have been biased by the lack of HLA-B*27 in the control group [17]. Neutrophils and monocytes from patients with active AAU have a decreased expression of Toll-like receptor 2 (TLR2), and reduced cytokine production on signaling through TLR4, which suggests a defect in response to a microbial trigger, and a defect in response to pathogens may be involved in AAU [18].

The lack of association between the SNPs of *PTPN22* and *CTLA-4* and AAU does not support dysfunction in the adaptive immune response. Therefore, the possibility exists that the defect is in the innate immune system and that AAU is autoinflammatory rather than autoimmune. Autoinflammation is described as local factors at sites predisposed to disease, leading to the activation of innate immune cells including macrophages and neutrophils [19]. Recent genetic studies endorse such a scenario. Polymorphisms in tumor necrosis factor (*TNF*; -308A and -238A), which are associated with increased production of the cytokine, are significantly more frequent in HLA-B*27–positive patients [20]. Similarly, HLA-B*27–positive patients with extraocular disease show an association with SNPs in the TNFRSF1A gene (*TNFR1*) compared to patients with AAU alone [21]. Interestingly, anti-TNF therapy has been shown to decrease the number of anterior chamber cells rapidly, to speed resolution of disease, and to reduce the rate of relapse on follow-up [22,23]. A SNP in the gene encoding *CCL-2* (−2518G), which is associated with significantly higher production of a chemokine involved in monocyte and neutrophil migration, was significantly associated with HLA-B*27–positive AAU [24]. Increased production and response to TNF in response to infection and exacerbated by the presence of HLA-B*27 [25]  would lead to endothelial activation and increased chemokine expression in many sites including the eye with the resultant influx of blood leucocytes, the majority of which would not be ocular specific. The self-limiting and relapsing nature of AAU could be explained by these mechanisms

However, other genes are involved in AAU. A recent whole genome SNP analysis comparing patients with AAU and patients with AS identified a region of chromosome 9 that was linked to AAU but not AS unlike most of the other hotspots [26]. The specific genes involved are currently being investigated.

*CTLA-4* polymorphisms have been reported in other ocular diseases. Similar to the findings in the current study, −318C/T and 49A/G were not significantly associated with intermediate uveitis or Behcet’s disease [13]. Conversely, in Chinese patients with Vogt-Koyanagi-Harada (VKH) syndrome 49G, and a haplotype based on four CTLA-4 SNPs, linked to reduced function, were associated with susceptibility to the syndrome [27]. In patients with Fuch's heterochromic cyclitis CTLA-4 -318T and a microsatellite repeat, linked to increased function, were associated with disease [28]. These differences may be explained by the nature of the disease type, autoimmune (VKH) or infectious (FHC), compared to idiopathic uveitis or possibly due to ethnic differences. These aspects should be investigated in future studies.

There are caveats in our study. The SNPs tested are not the only ones reported for these genes, and it remains possible that other mutations may influence AAU. Similarly, other genes including *NOD2* and *FOXP3* are regarded as master switches of autoimmunity, and these genes were not included in this study. In conclusion, the lack of association between SNPs in the genetic master switches of autoimmunity, *PTPN22* and *CTLA-4*, suggests that regardless of the strong linkage with HLA-B*27, AAU should be regarded as an autoinflammatory rather than an autoimmune condition.
